# The Dynamics of Arrivals of Maine Migratory Breeding Birds: Results from a 24-Year Study

**DOI:** 10.3390/biology6040038

**Published:** 2017-11-12

**Authors:** W. Herbert Wilson

**Affiliations:** Department of Biology, 5739 Mayflower Hill Drive, Colby College, Waterville, ME 04901, USA; whwilson@colby.edu; Tel.: +1-207-859-5739

**Keywords:** bird migration, phenology, climate change

## Abstract

This citizen-science project is the first systematic study of patterns of spring migration of Maine migratory birds. A comparison of arrival data from the Maine Ornithological Society from 1899–1911 with the modern data (1994–2017) collected for this study indicated that most species are now not arriving earlier, contrary to the predictions of earlier arrivals in the face of global warming. Arrival was synchronous across the lower two-thirds of the state for most species, although some species showed delayed arrivals along the northeastern coast compared to southern coastal areas. Only thirteen of 81 species are now arriving earlier and seven arriving later. Using quantile regression analysis with three levels of tau, the effect of year, temperature-departure from mean monthly temperature and the North Atlantic Oscillation Index were weak. Most species did not respond to any of these explanatory variables using the modern data. Leaf-gleaners showed the strongest responses. Only four species showed increasing abundance in recent years, an effect that influences detectability and hence could confound interpretation of changes in arrival date.

## 1. Introduction

The roots of the science of phenology can be traced back to the early 18th century in Britain and Sweden, with the first documentation of the arrivals and departures of British migratory birds [1,2]. These early phenological studies had practical applications, as farmers delayed the start of planting and other agricultural activities until particular birds arrived. The applied aspects of phenology are evident as a tool to monitor the impacts of global climate change [2,3,4,5].

The phenology of spring arrival dates and fall departure dates has contributed greatly to our understanding of the patterns of bird migration [6,7,8,9,10]. Birds therefore are powerful sentinels of environmental change [11].

In this contribution, I examine 24 years of data on spring arrival dates of migratory birds. This project was first conceived with the goal of elucidating the nature of spring migration in Maine. Maine spans a distance of 515 km from south to north, and varies in altitude from sea-level to 1606 m. Pronounced variation in plant phenology occurs along these gradients. The frost-free period in south coastal Maine is 170 days compared to only 80 days in northwestern Maine [12]. Balsam fir (*Abies balsamea*) growth in the spring is initiated 12 days earlier in southern Maine than in northwestern Maine [13]. It is therefore reasonable to expect that arrival of spring migrants will vary across the state.

Although two treatises on Maine birds have been published [14,15], no systematic study of bird migration in Maine has been undertaken. After the project was begun in 1994, it was evident that viewing the data through the lens of global climate change could also be profitable. With the aid of over 400 observers, the data from this citizen-science study serve the two-fold purpose of increasing our knowledge of patterns of spring migration in the state of Maine and assessing the impact of abiotic environmental characteristics on the arrival dates of those birds.

## 2. Materials and Methods

**Figure 1:**
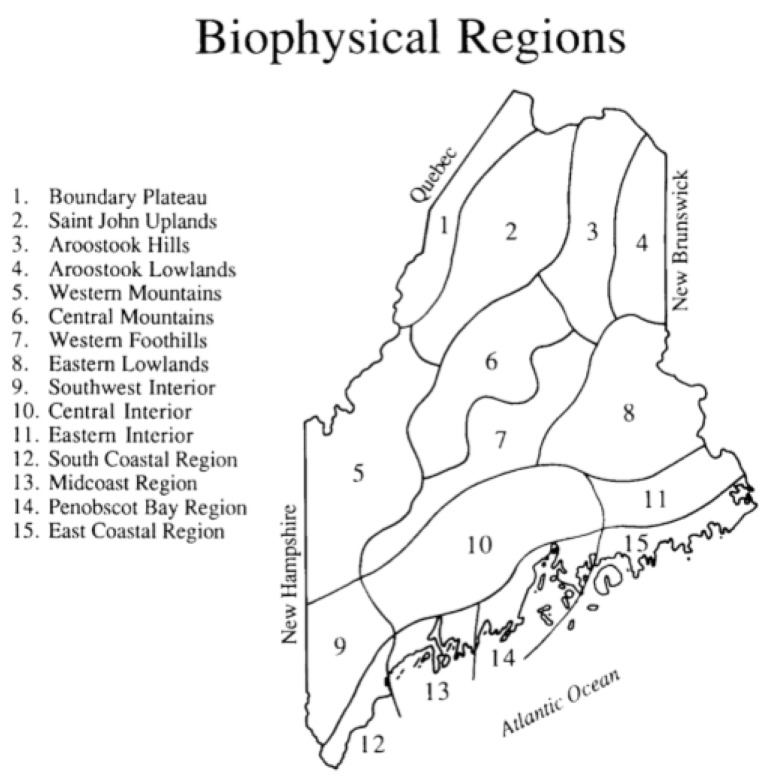
Maine Biophysical Regions, delineated on the basis of plant distributional data and climatic features [12].

The data for this project come primarily from volunteer observers across the state of Maine. Each volunteer was given an observation form and was asked to record the first arrival of any of 126 species of Maine migratory breeding birds. The observer also provided the geographic location of each sighting by identifying the Biophysical Region in which the sighting was made [12]. These regions (Figure 1) are based on a combination of meteorological data and plant distributional data and provide a convenient way to analyze spring phenological events. Some observers were active in more than one Biophysical Region, and thus contributed multiple first arrivals for some bird species. Arrival dates in January were eliminated because these records likely were overwintering birds rather than early spring arrivals.

The dataset consisted of 64,373 arrival dates for the 126 species. Over 400 observers contributed data over the course of the study.

To determine how the wave of migration spreads across the state of Maine, I used the programming language R to perform one-way analyses of variance (ANOVA) for each species to compare mean arrivals in different Biophysical Regions. Because of limited sample sizes for some regions (Table 1), I restricted the analysis to one central inland area (Region 10) and the four coastal regions (Region 12 along the southern coast heading northeastward to Region 15, terminating at the southern limit of the Bay of Fundy). If the ANOVA indicated that differences among arrivals in the five regions, I used the Tukey Honest Significant Difference (HSD) test to determine where the significant differences lay.

To compare arrival dates from the present study with historical records, I took advantage of the arrival dates reported in The Journal of the Maine Ornithological Society over the lifetime of the publication (1899–1911). My students and I extracted over 3000 first-of-year dates for 82 species. We combined the data for these years and compared them by species to the combined data of the modern data (1994–2017) using two-tailed, unpaired Student’s *t*-tests in R.

Three independent variables were tested for their power in explaining patterns of arrival for 112 species over the 24-year period. First, the year was used, with the prediction that arrivals would show a negative relationship with time, consistent with earlier arrivals as warming of the climate proceeded.

Second, the departure from average monthly temperature in each year was established as an independent variable. Because regional temperatures are correlated from Maine as far south as Delaware [16], migrating birds might adjust their arrival into Maine in the spring as a function of temperatures in the middle Atlantic and southern New England states. A warm spring in Delaware might induce birds to continue their northward migration and arrive relatively early in Maine. I used data from the National Climatic Research Center (https://www.ncdc.noaa.gov/) for measurements of temperature-departure from the mean monthly temperatures. I chose 12 weather stations in the southern two-thirds of Maine, averaging the departure of each station from the monthly average to yield a grand mean for each month.

The National Climatic Research Center only provides monthly temperature-departure data, precluding the use of time intervals tailored for the migration duration of each species. The month used for comparison depended on the mean arrival date of each species. For example, March temperature-departure data were used for Red-winged Blackbird (mean arrival of March 22) and May temperature-departure data for Swainson’s Thrush (mean arrival of May 19). The majority of first arrivals for each species occurred over a two-week interval. If the mean arrival date of a species was within six days of the end or beginning of a month, I used the mean of the two consecutive temperature-departure means. As an example, the mean of the April and May temperature-departures was used to analyze the arrival of Yellow-rumped Warblers (*Setophaga coronata)* whose mean arrival date is 28 April.

The strength of the North Atlantic Oscillation (NAO) can be used to predict the severity and snowiness of winter in northern North America and western Europe. NAO indices were taken from http://www.cpc.ncep.noaa.gov/data/teledoc/nao.shtml. A strong NAO is caused by a strong difference in air pressure between the persistent Icelandic Low and Azores High. High values of the NAO result in colder and drier winters in New England and Maritime Canada. Weak differences between the two air masses causes warmer and snowier weather in New England. Spring should arrive later in New England following winters with high NAO indices.

Quantile regression was used to test the effect of these three variables on arrival dates. Quantile regression is advantageous because one can detect patterns that are driven by particular quantiles of a distribution [17]. Under the expectation that arrivals should be earlier now, I chose to use values of tau (the particular quantile emphasized) of 0.1, 0.3 and 0.5. The low tau values test for effects of the hardiest birds, those arriving in advance of most of the remainder of the breeding population. A tau of 0.5 provides a balanced regression, similar to an Ordinary Least Squares Regression.

The analyses were performed in R using the quantreg package. The model used year, temperature-departure from mean and NAO Index as independent variables.

To search for possible relationships between foraging type on changing patterns of arrival, I classified the species into a feeding category [18]. These foraging categories are waterbirds, carrion-feeders, nectarivores, raptors, aerial insectivores, leaf-gleaning predators, ground-foraging predators, bark-gleaners, and seed-eaters.

Detectability of a particular species is influenced by abundance. Species that are declining may have later first arrivals recorded in more recent censuses that are a result of reduced opportunities for observing a first arrival rather than a change in patterns of migration. To control for this possible effect, I used population dynamics data from the North American Breeding Bird Survey (BBS) (https://www.mbr-pwrc.usgs.gov/bbs/) for Maine. The BBS provides two trend analyses, one for 1966–2015, and one for 2005–2015. I used 2005–2015 analyses because they are contained within the 1994–2017 interval of the modern data in this study.

In total, I performed 98 ANOVAs to examine the arrivals of birds among five Biophysical Regions, 81 t-tests to compare historic versus recent arrival dates, and 112 quantile regressions to examine the impact of spring-time temperature, year and NAO Index on arrival date. These multiple comparisons greatly elevate the likelihood of Type I errors. To control for the multiple comparisons, I used the Benjamini-Hochberg test to modify the individual *p*-values to account for false discoveries and produce an experiment-wise alpha of 0.05 for each battery of tests [19]. In all cases, an individual p-value less than 0.0001 was required to claim a significant difference for a particular test. The Benjamini-Hochberg tests were performed in R with the bh-test command.

## 3. Results

The intensity of coverage varied both temporally and spatially, an unavoidable consequence of a citizen-science project. The mean number of observations per year was 2682, with a high of 5354 in 1994 (the first year of the project) and a low of 1429 in 2014. Cumulative means for each species within a year converged within a day or two of the sample mean once 12 to 15 dates were added to the analysis. The sample size of nearly all the species-year combinations exceeded 12 observations, frequently exceeding 30.

The intensity of the effort in each Biophysical Region (Table 1) was a reflection of human population density. The best sampled region was Region Ten, including the populous cities of Lewiston/Auburn, Augusta, Waterville and Bangor. The four coastal regions were also well sampled. The four northernmost Regions and Region Six (including Baxter State Park) were poorly covered. The earliest records for Region One come from 2006.

**Figure 2:**
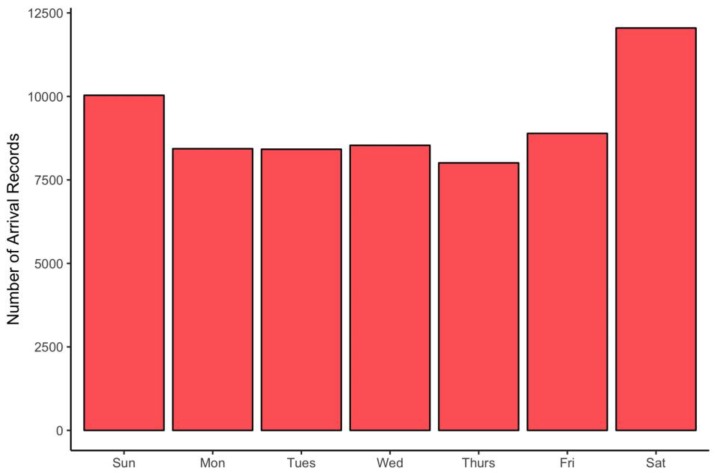
Frequency of observations by day of week.

The expectation that more observations would be recorded on weekends was borne out by the data (Figure 2) although the bias toward weekend observations is relatively modest.

Forty-two species showed differences in arrival dates across the five best-sampled Biophysical Regions (Table 2). Sixteen species arrived significantly later in Region 15, the northernmost coastal region. One species arrived earlier in Region 12 and seven species arrived earlier in Regions 12 and 13. Three species arrived later in Region 10 than the other species. The remaining 13 species showed more complex patterns but the general trend is for later arrivals in Region 15. Fifty-six species showed synchronous arrivals across the five Biophysical Regions.

Table 3 presents the data comparing the arrivals of birds between the period 1899-1911 and the 24-year modern data set. Sufficient data were available to compare 81 species. The most common result was no change in arrival dates between the two time periods; 61 species showed no significant change in mean arrival date. Thirteen of the remaining 20 species are arriving earlier now than in the earlier period. Seven species show the opposite of the expected pattern, arriving later now than at the turn of the 20th century.

**Figure 3:**
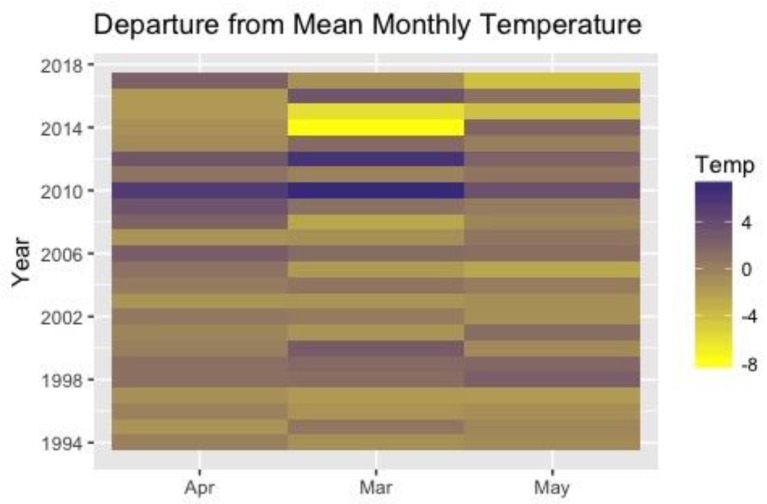
Heat map of departure from mean of spring-time monthly temperatures over the course of the study. Temperature is given in °F.

Figure 3 shows the patterns of departure of monthly temperature from the mean over the course of the 24-year study. Monthly temperature varies widely from year-to-year for a single month and within a year. In other words, there is no monotonic change in temperature over the 24 years of this project. Within a year, the temperature-departure of March, April and May often vary widely in magnitude and direction. A warm March does not predict a warm April or warm May.

Table 4 presents the results of the quantile regression analyses. When tau is set to 0.1 (emphasizing the first decile of the data) in the quantile regression model, eight of the 112 species showed the predicted negative relationship with time and two species showed an anomalous positive relationship. Twelve species showed a negative relationship with temperature-departure from the mean and only one showed a positive relationship. The NAO Index had a weaker effect with two species showing the expected negative relationship and two showing a positive relationship.

With tau set to 0.3, fifteen species showed a pattern of earlier arrival over the 24 years of the study with a single species showing the opposite pattern. Fourteen species arrived earlier in warm springs with only one showing earlier arrivals in cold springs. Five species arrived earlier when the NAO Index was high and three arrived later when the NAO was high. 

With a balanced regression (tau = 0.5), 14 species show earlier arrival over the course of the study with only one showing the opposite pattern. Ten species arrived earlier in warm springs with none showing the opposite pattern. Three species showed arrivals negatively correlated with the NAO Index and none showed a positive relationship.

In overview, 37 of the 126 species analyzed showed a significant relationship of mean arrival date with time, temperature-departure from the mean or the NAO Index for at least one level of tau. The most common result was that none of the three independent variables explained arrival date.

A few taxonomic patterns are evident (Table 4). Only one of the 19 aerial insectivores showed any significant relationship with any of the three explanatory variables. Two of the five vireonids, nine of the 24 parulids, four of the nine sparrows and four of the seven blackbirds had arrival dates influenced by at least one of the three explanatory variables.

Four species are increasing in Maine based on Breeding Bird Survey data (Table 4). Such species could demonstrate spurious patterns of earlier arrival because of increasing detectability. Turkey Vultures showed significant negative relationships with Year, TempDep, and NAO in six of nine cases, so that population dynamics rather than actual changes in migratory arrival may operate in this case. The other three species that were increasing (Green Heron, Upland Sandpiper and Yellow-bellied Sapsucker) did not have arrival dates related to time or environmental variables.

Nineteen species are showing population decreases over the 2005–2015 interval (Table 4). The decrease in detectability over the course of this project will tend to reduce the detection of earlier arrivals and hence provide more conservative results. Species that show consistent but non-significant effects of time, temperature departure from the mean, and NAO Index, include Tree Swallow, Bank Swallow, Barn Swallow, Winter Wren, Northern Water thrush, Vesper Sparrow, Song Sparrow, White-throated Sparrow, and Bobolink.

## 4. Discussion

This project is the first comprehensive effort to understand the patterns of spring bird migration in Maine. With mountains along much of the western border of the state and with more moderate temperatures along the coast, I predicted that birds would likely either arrive first along the coast and spread westward, ultimately reaching the mountains, or that birds would arrive in southern Maine and then fan out, again reaching the mountainous regions last. I also expected a latitudinal effect with birds arriving in southern Maine before reaching more northerly sites.

Based on the first four years of the project [6], patterns of arrival across the 15 Biophysical Regions of the state (Figure 1) were examined. With the exception of a few species that arrived later across the northern tier of the state (Regions 1–4), few differences in arrival emerged. The statistical power of these comparisons was limited by sample size in many cases, increasing the likelihood of Type II errors.

To increase the power of the tests, I repeated the analyses using the full 24-year dataset. Only the five best sampled Biophysical Regions were modeled (Table 1 and Table 2). ANOVAs indicated that arrivals were synchronous for the majority of the species (Table 2). However, 42 species did show significant differences among Biophysical Regions. The prevalent patterns of those 42 species was either a later arrival in Region 15 or earlier arrival in Region 12 or 12/13, consistent with the prediction of a south-to-north spread along the coast. We must remain agnostic on altitudinal effects on migration because of lack of sufficient data for mountainous Biophysical Regions.

Some of the first claims of earlier spring arrivals in recent years for eastern North America come from bird clubs or dedicated naturalists who compiled arrival dates every year. Analysis of records from 1903 to 1993 from the Cayuga Bird Club in upstate New York and from 1932 to 1993 from the Worcester County Ornithological Society in central Massachusetts found that all 103 species examined are arriving earlier now [20]. The change varied from 11 days for short-distance migrants to four days for Neotropical migrants. Similar patterns were documented in southeastern Massachusetts and Duchess County, New York. Five of 16 bird species at a site in eastern Massachusetts show a pattern of earlier arrivals from 1970 until 2002 [21]. In Duchess County, New York, data from the records of Ralph C. Waterman Bird Club from 1885 to 2008 and field notes of local birders for 44 species of migratory breeding birds provided the basis of an analysis [22]. Forty of the 44 species showed earlier arrivals in recent years.

Although no such continuous record of arrival dates exists for Maine, the records published in the Journal of the Maine Ornithological Society (1899–1910) provide a yardstick for comparison with the recent 24-year dataset [23]. The most common result for 82 species was no change in arrival date between the two time-periods (Table 2). Thirteen species are arriving earlier now than at beginning of the 20th century, conforming to the prediction of earlier arrival in the face of global warming. Anomalously, seven species demonstrate later arrival dates in recent years. Three of these species (Eastern Bluebird, Savannah Sparrow, Vesper Sparrow) are grassland birds. We know that grasslands and agricultural acreage was much greater in 1900 than today [24], and hence that populations of these species were likely much larger. A higher likelihood of encountering a first-arrival early should vary directly with population size. Two of the later-arriving species (Whip-poor-will and Common Night-Hawk) may have been more abundant a century ago [14,15] and hence more likely to be detected early than in 1900. I am at a loss to understand the reasons for later current arrivals of Chimney Swift and Least Flycatcher.

The failure to find consistent increasingly early arrival dates in Maine contrasts with the data for Massachusetts and New York [20,21,22]. Part of this explanation is that the three prior studies did not control for multiple comparisons to produce experiment-wise alpha values of 0.05. Failure to correct for multiple comparisons leads to false positive results. However, the magnitude of the significant differences observed (often in excess of 10 days) exceeds the significant changes seen for Maine arrivals (Table 3). The results of the current study are more consistent with the analysis of banding data from Long Point Bird Observatory [25] where only two of 13 species showed earlier arrivals over the period of 1975 to 2000.

The data from this project allow testing of patterns of change of arrival dates correlated with time, temperature-departure from the mean for the month of migration and the NAO Index over the period from 1994 through 2017. Premised on the effect of global warming, each of these three explanatory variables should be negatively related to the mean arrival date.

Because natural selection should be favoring early arrivals as the climate warms [16,26], I used quantile regression to allow the exploration of the bold, earliest arriving birds by biasing the analysis in favor of the left side of the distribution of arrival dates. With tau = 0.1, only eight species are arriving earlier within the 1994–2017 interval (Table 4). With tau = 0.3, 15 species show a negative slope and 14 species show a negative slope when tau = 0.5. However, the dominant pattern is for no change to be happening for the 112 species analyzed.

The 24-year span of this project should be adequate to detect modern changes in arrival date, because some species in New York began to arrive earlier over a 27-year period [27]. If birds are timing their arrival to temperature, one should see a relationship between arrival data and temperature-departure from the mean temperature for the month of migration. Temperature-departure data are strongly correlated from Maine to Maryland and Delaware [16]. The dominant pattern is no relationship between temperature-departure and arrival date (Table 4). Only twelve, fourteen and ten species showed a significant relationship in quantile regressions with tau set to 0.1, 0.3, and 0.5 respectively. 

Weak effects emerged for the NAO Index (Table 4). The overwhelming result was that the NAO Index does not influence arrival. Only two, five and three species out of 112 showed a significant negative relationship in quantile regressions with tau set to 0.1, 0.3 and 0.5 respectively.

Population dynamics may influence the power of this analysis. Four species are increasing with Turkey Vulture showing strong negative effects (Table 4) for all three variables. At least some of the explanation may be due to the increase in the size of the Turkey Vulture population.

Nineteen species are declining in abundance, making it more difficult to detect earlier arrivals because of reduced encounter opportunities in recent years. Nine of these species show non-significant trends of time and environmental variables on arrival suggesting that population dynamics may be masking a response to climate change.

## 5. Conclusions

In summary, the data from this citizen-science project indicate about half of the species analyzed show arrival patterns across the state that are consistent with patterns of spring temperature increase along a latitudinal gradient (Table 2). The impacts of global warming on Maine spring bird migration are modest to date. Fewer than 16% of species analyzed show an earlier arrival now than in the period 1899–1910. Within the past 24 years, only a minority of species respond to temperature-deviations from the norm in the spring and to the NAO Index. Although the reality of global warming is clear [1,2,3,4], avian responses may be slow to occur because migratory schedules are hard-wired rather than reactive [28]. If so, avian responses to climate change may require many years to evolve.

## Figures and Tables

**Table 1:** Observations by Biophysical Region.

Biophysical Region	Number of Observations
Ten	19,895
Twelve	14,213
Thirteen	11.226
Fourteen	5182
Fifteen	4721
Nine	2434
Seven	2018
Eleven	1713
Eight	1451
Five	861
One	372
Six	128
Two	79
Four	42
Three	38

**Table 2:** Comparison of mean arrival dates in the five best sampled Biophysical Regions (see Table 1). Benjamini-Hochberg corrections were used to adjust for false discovery rates because of multiple comparisons [17]. Asterisks indicate significant differences (*p* < 0.05) after correction. Dates with the same letter are not significantly different from each other in Tukey Honest Significant Difference pair-wise tests. Species order follows the North American Classification Committee Checklist of North American Birds (http://checklist.aou.org/).

Species	Ten	Twelve	Thirteen	Fourteen	Fifteen	Significance
Wood Duck (*Aix sponsa*)	4/5 a	4/8 a	4/8 a	4/11 b	4/15 b	NS
Green-winged Teal (*Anas crecca*)	4/6 a	4/1 ab	3/30 b	4/8 bc	4/16 c	*
Ring-necked Duck (*Aythya collaris*)	4/3 a	3/26 b	3/29 ab	4/2 ab	4/13 c	*
Pied-billed Grebe (*Podilymbus podiceps*)	4/18 ab	4/8 b	4/16 ab	4/28 a	4/15 ab	*
Black-billed Cuckoo (*Coccyzus erythropthalmus*)	5/30 a	5/25 a	5/23 a	5/25 a	NA	NS
Common Nighthawk (*Chordeiles minor*)	5/24 a	5/21 a	5/23 a	5/23 a	5/28 a	NS
Eastern Whip-poor-will (*Caprimulgus vociferus*)	5/24 a	5/11 a	5/15 a	5/21 a	5/25 a	NS
Chimney Swift (*Chaetura pelagica*)	5/11 a	5/11 a	5/13 ab	5/12 ab	5/18 b	*
Ruby-throated Hummingbird (*Archilochus colubris*)	5/12 a	5/9 b	5/10 b	5/9 b	5/11 ab	*
Virginia Rail (*Rallus limicola*)	5/9 a	5/8 a	5/12 a	5/4 a	5/10 a	NS
Sora (*Porzana carolina*)	5/12 a	5/13 a	5/12 a	4/30 a	5/17 a	NS
Wilson’s Snipe (*Gallinago delicata*)	4/19 a	4/18 a	4/13 a	4/24 a	4/28 a	NS
American Woodcock (*Scolopax minor*)	4/2 a	3/28 a	3/30 a	3/27 a	3/31 a	NS
Spotted Sandpiper (*Actitis macularia*)	5/9 a	5/11 a	5/12 a	5/18 b	5/17 b	*
Least Tern (*Sterna antillarum*)	NA	5/22 a	5/27 a	5/28 a	NA	NS
Common Tern (*Sterna hirundo*)	NA	5/18 a	5/21 a	5/20 a	5/27 a	NS
Arctic Tern (*Sterna paradisaea*)	NA	5/31 a	5/24 a	5/30 a	5/31 a	NS
Common Loon (*Gavia immer*)	4/18 a	4/18 a	4/11 a	4/14 a	4/28 a	NS
American Bittern (*Botaurus lentiginosus*)	4/29 a	4/26 a	4/25 a	4/25 a	5/4 a	NS
Great Blue Heron (*Ardea herodias*)	4/14 a	4/5 b	4/5 b	4/14 a	4/16 a	*
Turkey Vulture (*Catharus aura*)	3/29 a	3/17 b	3/21 ab	3/24 a	4/10 c	*
Osprey (*Pandion haliaetus)*	4/14 a	4/16 ac	4/8 b	4/14 a	4/21 c	*
Broad-winged Hawk (*Buteo platypterus*)	4/25 a	4/26 a	4/21 a	4/25 a	4/29 a	NS
Belted Kingfisher (*Megaceryle torquatus*)	4/24 a	4/12 b	4/13 b	4/20 a	4/25 a	*
Yellow-bellied Sapsucker (*Sphyrapicus varius*)	4/20 a	4/17 a	4/16 a	4/24 a	4/21 a	NS
Northern Flicker (*Colaptes auratus*)	4/16 a	4/5 b	4/9 b	4/11 b	4/16 ab	*
American Kestrel (*Falco sparverius*)	4/3 a	4/6 ab	4/7 b	4/4 ab	4/14 c	*
Olive-sided Flycatcher (*Contopus cooperi*)	5/24 a	5/24 a	5/22 a	5/25 a	5/23 a	NS
Eastern Wood-Pewee (*Contopus virens*)	5/24 ab	5/22 a	5/21 a	5/27 b	5/28 b	*
Yellow-bellied Flycatcher (*Empidonax flaviventris*)	5/25 a	5/24 a	5/21 a	5/24 a	5/21 a	NS
Alder Flycatcher (*Empidonax alnorum*)	5/24 a	5/25 a	5/23 a	5/23 a	5/25 a	NS
Willow Flycatcher (*Empidonax trailii*)	5/24 a	5/27 a	5/27 a	5/24 a	5/24 a	NS
Least Flycatcher (*Empidonax minimus*)	5/12 a	5/14 a	5/14 a	5/16 a	5/25 a	NS
Eastern Phoebe (*Sayornis phoebe*)	4/9 a	4/7 a	4/8 a	4/6 a	4/16 b	*
Great Crested Flycatcher (*Myiarchus crinitus*)	5/13 a	5/11 a	5/13 a	5/14 a	5/22 b	*
Eastern Kingbird (*Tyrannus tyrannus*)	5/11 ab	5/12 a	5/14 ab	5/12 b	5/19 c	*
Philadelphia Vireo (*Vireo pheladelphia*)	5/22 a	5/19 a	5/17 a	5/24 a	5/25 a	NS
Warbling Vireo (*Vireo gilvus*)	5/12 a	5/14 a	5/13 a	5/19 a	5/16 a	NS
Red-eyed Vireo (*Vireo olivaceus*)	5/18 a	5/16 a	5/18 a	5/19 a	5/26 b	*
Purple Martin (*Progne subis*)	5/9 a	5/6 a	5/4 a	4/29 a	5/8 a	NS
Tree Swallow (*Tachycineta bicolor*)	4/13 a	4/13 a	4/15 a	4/16 a	4/25 b	*
Bank Swallow (*Riparia riparia*)	5/14 a	5/16 a	5/13 a	5/13 a	5/15 a	NS
Cliff Swallow (*Hirundo pyrrhonota*)	5/11 a	5/15 a	5/10 a	5/13 a	5/19 a	NS
Barn Swallow (*Hirundo rustica*)	5/3 a	5/4 a	5/2 a	5/6 a	5/7 a	NS
House Wren (*Troglodytes aedon*)	5/8 a	5/6 a	5/9 a	5/12 a	5/18 a	NS
Winter Wren (*Troglodytes troglodytes*)	4/24 a	4/23 a	4/20 a	4/22 a	4/22 a	NS
Blue-gray Gnatcatcher (*Polioptila caerulea*)	5/6 a	5/3 a	5/4 a	5/6 a	5/7 a	NS
Ruby-crowned Kinglet (*Regulus calendula*)	4/23 a	4/22 a	4/21 a	4/20 a	4/25 a	NS
Eastern Bluebird (*Sialia sialis*)	4/10 a	4/2 a	4/8 a	4/17 a	5/11 a	NS
Veery (*Catharus fuscescens*)	5/15 a	5/13 a	5/14 a	5/17 a	5/20 a	NS
Swainson’s Thrush (*Catharus ustulatus*)	5/21 a	5/15 b	5/16 b	5/16 b	5/25 a	*
Hermit Thrush (*Catharus guttatus*)	4/23 a	4/22 a	4/21 a	4/23 a	4/26 a	NS
American Robin (*Turdus migratorius*)	3/18 a	3/15 a	3/15 a	3/17 a	3/26 b	*
Gray Catbird (*Dumatella carolinensis*)	5/11 a	5/6 b	5/7 b	5/12 a	5/13 a	*
Brown Thrasher (*Toxostoma rufum*)	5/7 a	5/7 a	5/8 a	5/7 a	5/13 a	NS
Ovenbird (*Seiurus aurocapilla*)	5/8 a	5/8 a	5/8 a	5/8 a	5/12 b	*
Louisiana Waterthrush (*Parkesia motacilla*)	5/7 a	5/5 a	5/1 a	5/15 a	NA	NS
Northern Waterthrush (*Parkesia novaeboracensis*)	5/8 a	5/11 b	5/7 a	5/8 a	5/19 c	*
Black-and-white Warbler (*Mniotilta varia*)	5/5 a	5/4 a	5/4 a	5/5 a	5/10 b	*
Tennessee Warbler (*Leiothlypis peregrina*)	5/18 ac	5/18 ac	5/19 a	5/22 a	5/13 a	NS
Nashville Warbler (*Leiothlypis ruficapilla*)	5/8 a	5/9 a	5/8 a	5/9 a	5/12 a	NS
Mourning Warbler (*Geothlypis philadelphia*)	5/24 a	5/21 a	5/24 a	5/21 a	5/28 a	NS
Common Yellowthroat (*Geothlypis trichas*)	5/11 a	5/10 a	5/11 a	5/12 a	5/19 a	NS
American Redstart (*Setophaga ruticilla*)	5/14 ab	5/12 a	5/14 ab	5/17 b	5/21 c	*
Cape May Warbler (*Setophaga tigrina*)	5/13 a	5/16 a	5/15 AA	5/19 a	5/17 a	NS
Northern Parula (*Setophaga parula*)	5/8 a	5/8 a	5/7 a	5/8 a	5/10 a	NS
Magnolia Warbler (*Setophaga magnolia*)	5/14 a	5/14 a	5/13 a	5/16 a	5/19 b	*
Bay-breasted Warbler (*Setophaga castanea*)	5/18 a	5/16 a	5/17 a	5/17 a	5/22 a	NS
Blackburnian Warbler (*Setophaga fusca*)	5/15 a	5/14 a	5/15 a	5/14 a	5/20 b	*
Yellow Warbler (*Setophaga petchia*)	5/10 a	5/9 a	5/11 a	5/12 ab	5/16 b	*
Chestnut-sided Warbler (*Setophaga pensylvanica*)	5/12 a	5/12 a	5/11 a	5/13 a	5/20 b	*
Blackpoll Warbler (*Setophaga striata*)	5/22 a	5/18 b	5/17 b	5/20 ab	5/21 ab	*
Black-throated Blue Warbler (*Setophaga caerulescens*)	5/12 a	5/10 a	5/11 a	5/10 a	5/19 b	*
Palm Warbler (*Setophaga palmarum*)	4/23 a	4/22 a	4/20 a	4/21 a	4/22 a	NS
Pine Warbler (*Setophaga pinus*)	4/25 a	4/22 a	4/23 a	4/28 a	4/29 a	NS
Yellow-rumped Warbler (*Setophaga coronata*)	4/27 a	4/28 a	4/25 a	4/27 a	5/1 a	NS
Prairie Warbler (*Setophaga discolor*)	5/10 a	5/13 a	5/12 a	5/15 a	NA	NS
Black-throated Green Warbler (*Setophaga virens*)	5/7 a	5/5 a	5/5 a	5/6 a	5/10 b	*
Canada Warbler (*Cardellina canadensis*)	5/21 a	5/17 b	5/16 b	5/21 a	5/20 a	*
Wilson’s Warbler (*Cardellina pusilla*)	5/16 a	5/15 a	5/15 a	5/16 a	5/19 a	NS
Chipping Sparrow (*Spizella passerina*)	4/22 a	4/17 a	4/20 a	4/25 ac	4/27 a	NS
Field Sparrow (*Spizella pusilla*)	4/30 a	5/4 a	5/1 a	5/5 a	NA	NS
Vesper Sparrow (*Chondestes gramineus*)	4/30 a	5/9 b	4/23 a	4/27 a	5/3 ab	*
Savannah Sparrow (*Passerculus sandwichensis*)	4/23 a	4/22 a	4/23 a	4/24 a	4/29 a	NS
Fox Sparrow (*Passerella iliaca*)	4/5 a	3/25 b	3/26 b	3/26 b	3/28 b	*
Song Sparrow (*Melospiza melodia*)	3/29 a	3/21 b	3/22 b	3/23 b	4/12 a	*
Lincoln’s Sparrow (*Melospiza lincolni*)	5/12 a	5/12 a	5/9 a	5/11 a	5/13 a	NS
Swamp Sparrow (*Melospiza georgiana*)	4/28 a	4/27 a	4/26 a	5/1 a	5/3 a	NS
White-throated Sparrow (*Zonotrichia albicollis*)	4/19 a	4/9 b	4/14 b	4/19 a	4/16 a	*
Scarlet Tanager (*Piranga olivacea*)	5/17 a	5/14 a	5/17 ab	5/18 ab	5/22 b	*
Rose-breasted Grosbeak (*Pheucticus ludovicianus*)	5/9 a	5/9 a	5/10 a	5/12 ab	5/13 b	*
Indigo Bunting (*Passerina cyanea*)	5/21 a	5/18 ab	5/16 b	5/15 b	5/13 b	*
Bobolink (*Dolichonyx oryzivorus*)	5/12 a	5/15 b	5/15 b	5/17 c	5/21 c	*
Red-winged Blackbird (*Aegelaius phoeniceus*)	3/31 a	3/27 a	3/28 a	3/25 a	3/29 a	NS
Rusty Blackbird (*Euphagus carolina)*	4/14 a	4/8 a	4/3 a	4/9 a	4/10 a	NS
Brown-headed Cowbird (*Molothrus ater*)	4/6 a	3/31 a	3/29 a	3/26 a	4/16 a	NS
Common Grackle (*Quiscalus quiscala*)	3/23 a	3/18 a	3/20 a	3/24 a	4/2 b	*
Baltimore Oriole (*Icterus galbula*)	5/5 a	5/5 a	5/3 a	5/30 a	5/8 a	NS

**Table 3:** Comparison of mean arrival date between the early period of 1899–1911 (data from Journal of Maine Ornithological Society) and the recent period of 1994–2017 using the present data. To control for false discovery rates because of multiple comparisons, the Benjamini-Hochberg corrected was applied [17]. Asterisks indicate significant differences (*p* < 0.05) after correction.

Species	1899–1911	1994–2017	*p*
Wood Duck	4/15	4/8	NS
Pied-billed Grebe	4/9	4/18	NS
Black-billed Cuckoo	5/25	5/27	NS
Common Nighthawk	5/19	5/26	*
Whip-poor-will	5/10	5/19	*
Chimney Swift	5/9	5/15	*
Ruby-throated Hummingbird	5/18	5/11	*
Wilson’s Snipe	4/23	4/20	NS
American Woodcock	4/13	3/30	*
Spotted Sandpiper	5/9	5/11	NS
Common Loon	5/9	4/19	*
American Bittern	5/8	5/2	NS
Great Blue Heron	4/22	4/12	*
Broad-winged Hawk	4/26	4/26	NS
Belted Kingfisher	4/25	4/20	*
Yellow-bellied Sapsucker	4/23	4/20	NS
Northern Flicker	4/14	4/14	NS
Olive-sided Flycatcher	5/22	5/26	NS
Eastern Wood-Pewee	5/23	5/22	NS
Yellow-bellied Flycatcher	5/25	5/22	NS
Alder Flycatcher	5/23	5/26	*
Least Flycatcher	5/10	5/14	*
Eastern Phoebe	4/6	4/9	*
Great Crested Flycatcher	5/17	5/14	*
Eastern Kingbird	5/15	5/15	NS
Yellow-throated Vireo	5/20	5/19	NS
Blue-headed Vireo	5/9	5/3	*
Philadelphia Vireo	5/25	5/13	NS
Warbling Vireo	5/17	5/15	NS
Red-eyed Vireo	5/22	5/18	*
Purple Martin	5/6	5/10	*
Tree Swallow	4/20	4/16	*
Bank Swallow	5/12	5/17	NS
Cliff Swallow	5/10	5/15	*
Barn Swallow	5/3	5/6	*
Winter Wren	4/18	4/23	NS
Ruby-crowned Kinglet	4/25	4/22	NS
Eastern Bluebird	3/23	4/11	*
Veery	5/12	5/17	NS
Hermit Thrush	4/18	4/26	*
American Robin	3/30	3/29	*
Gray Catbird	5/15	5/9	*
Brown Thrasher	5/11	5/9	*
Ovenbird	5/12	5/10	*
Northern Waterthrush	5/11	5/9	NS
Black-and-white Warbler	5/9	5/7	NS
Tennessee Warbler	5/26	5/18	*
Nashville Warbler	5/9	5/9	NS
Mourning Warbler	5/25	5/26	NS
Common Yellowthroat	5/14	5/12	*
American Redstart	5/15	5/17	*
Northern Parula	5/11	5/10	*
Magnolia Warbler	5/15	5/14	NS
Bay-breasted Warbler	5/18	5/18	NS
Blackburnian Warbler	5/14	5/17	NS
Yellow Warbler	5/15	5/11	*
Chestnut-sided Warbler	5/17	5/15	*
Blackpoll Warbler	5/20	5/19	NS
Black-throated Blue Warbler	5/15	5/12	NS
Pine Warbler	4/26	4/26	NS
Yellow-rumped Warbler	4/28	4/28	NS
Black-throated Green Warbler	5/10	5/9	NS
Canada Warbler	5/18	5/19	NS
Wilson’s Warbler	5/18	5/16	*
Chipping Sparrow	4/23	4/21	*
Field Sparrow	4/28	5/3	*
Vesper Sparrow	4/16	5/2	*
Savannah Sparrow	4/19	4/26	*
Fox Sparrow	4/3	3/30	*
Song Sparrow	3/27	3/30	*
Lincoln’s Sparrow	5/3	5/12	NS
Swamp Sparrow	4/23	5/1	NS
White-throated Sparrow	4/27	4/16	*
Scarlet Tanager	5/19	5/17	*
Rose-breasted Grosbeak	5/16	5/10	*
Indigo Bunting	5/23	5/18	*
Bobolink	5/15	5/17	*
Red-winged Blackbird	4/3	3/20	*
Rusty Blackbird	4/3	4/11	*
Baltimore Oriole	5/17	5/7	*

**Table 4:** Results of quantile regressions testing the effect of time (Year) on arrival dates. The data presented are the regression coefficients for each independent variable. The Benjamini-Hochberg adjustment was used to correct for false discovery rates. Asterisks indicate a significant effect (*p* < 0.05) after correction. Data are given for three sets of quantile regressions with tau set to 0.01, 0.03 and 0.05. Foraging types are based on Ehrlich and Dobkin (1975). Key to abbreviations: AI—aerial insectivore, BG—bark gleaner, GF—ground feeder, LG—foliage gleaner, RA—raptor, NE—nectar or sap feeder, SE—seed eater, WB—waterbird [18]. PopChng gives the annual trend line of a species in Maine over the period 2005–2015 from the Breeding Bird Survey (https://www.mbr-pwrc.usgs.gov/bbs/). A value of 0 indicates the confidence interval for the analysis overlaps zero, and hence a claim of no population change over the interval cannot be rejected.

Species	MeanArr	n	Year	tau = 0.1	NAO	Year	tau = 0.3	NAO	Year	tau = 0.5	NAO	Foraging	PopChng
				TempDep			TempDep			TempDep			
Wood Duck	4/8	620	−0.52	−1.44	−0.66	−0.32	−1.50	−0.45	−0.29	−1.41	−1.00	WB	0
Blue-winged Teal	4/20	269	−0.02	−0.54	−0.12	−0.26	−0.81	0.52	−0.18	−1.11	−0.01	WB	0
Green-winged Teal	4/5	469	−0.34	−1.77	−0.86	−0.04	−1.83	−0.46	−0.37 *	−1.80	−0.39	WB	0
Ring-necked Duck	4/3	564	−0.74 *	−1.34 *	−0.91	−0.43 *	−1.6 *	−1.18	−0.45	−1.79	−1.64	WB	0
Pied-billed Grebe	4/18	259	0.04	0.50	−0.07	−0.09	−0.90	−0.23	−0.13	−0.83	0.05	WB	0
Black-billed Cuckoo	5/27	187	−0.11	−1.13	0.49	−0.17	−0.90	0.37	0.05	−0.82	0.45	FG	0
Common Nighthawk	5/25	271	−0.24	1.09	−0.41	−0.04	0.46	−0.03	−0.15	−0.34	−0.04	AI	0
Whip-poor-will	5/19	146	−0.53	3.52	−0.30	0.22	−0.29	−0.21	−0.03	0.09	−0.01	AI	0
Chimney Swift	5/13	580	0.07	−0.67	−0.10	0.10	−0.21	−0.11	0.08	−0.06	−0.50	AI	−5.82
Ruby-throated Hummingbird	5/11	1108	−0.24 *	0.28	0.50	−0.21 *	−0.22	0.12	−0.23 *	−0.07	−0.06	NE	0
Virginia Rail	5/10	209	−0.07	−0.83	−0.74	0.24	−1.96	0.03	0.53	−1.33	0.28	WB	0
Sora	5/12	207	0.18	0.15	0.04	−0.01	0.34	0.60	0.06	1.04	1.07	WB	0
Piping Plover	4/25	138	0.61	−0.41	−0.81	0.75	2.16	−0.91	0.64	2.93	0.29	WB	0
Killdeer	4/3	877	−0.13	−1.00	−1.04	−0.03	−1.22 *	−0.59	0.21	−0.66	−0.68	WB	0
Upland Sandpiper	5/12	143	−0.13	0.78	0.19	0.13	2.47	0.25	0.49	0.85	−0.56	WB	3.89
Wilson’s Snipe	4/21	389	−0.06	−0.67	−0.29	0.01	−0.99	−0.43	−0.03	−0.56	−0.58	WB	0
American Woodcock	3/31	777	−0.28 *	−1.59	−0.45 *	−0.23 *	−1.63	−0.71 *	−0.15 *	−1.43	−1.1 *	GF	0
Spotted Sandpiper	5/12	454	−0.14	−0.50	−0.21	−0.07	−0.27	0.00	−0.04	−0.23	−0.19	WB	0
Willet	5/6	240	−0.08	−0.96	0.19	0.00	0.16	0.35	−0.13	−0.18	−0.12	WB	0
Least Tern	5/24	97	−0.1	−0.65	0.14	0.02	−0.15	−0.04	0.14	−1.02	−0.27	WB	0
Black Tern	5/20	104	−0.43	0.32	−1.50	−0.22	−0.03	−0.67	−0.23	−0.49	−0.08	WB	0
Common Tern	5/21	246	−0.01	−0.23	−0.21	0.00	0.00	0.00	0.02	−0.37	0.00	WB	0
Common Loon	4/20	457	−0.2	−1.77 *	0.02	−0.13	−1.33	0.28	−0.03	−0.31	0.38	WB	0
American Bittern	4/30	356	0.05	0.11	1.02	0.20	−0.02	−0.71	0.15	1.10	−0.32	WB	0
Great Blue Heron	4/12	974	−0.01	−0.07	−0.47	−0.08	0.20	−0.49	−0.26 *	−0.14	−1.08	WB	0
Green Heron	5/11	247	0.11	0.36	0.37	−0.17	0.75	0.81	−0.10	0.29	−0.09	WB	−6.87
Black-crowned Night-Heron	5/2	189	−0.6	−1.03	−0.29	−0.53	0.23	0.72	−0.47	0.81	2.14	WB	0
Glossy Ibis	4/21	228	−0.05	0.34	0.16	0.12	0.22	0.44	0.07	−0.37	−0.18	WB	0
Turkey Vulture	3/26	1136	−0.67 *	−1.87 *	−0.78	−0.57 *	−1.37 *	−0.65	−0.62 *	−1.31 *	−0.80	CA	3.79
Osprey	4/15	955	−0.03	−0.23	−0.24	−0.27 *	−0.54	−0.35	−0.31 *	−0.50	−0.37	WB	0
Northern Harrier	4/9	683	−0.5	−0.51	−1.01	−0.25	−1.12	−0.68	−0.12	−0.68	−0.52	RA	0
Broad-winged Hawk	4/25	751	0.02	0.04	0.32	−0.70	0.46	0.14	−0.03	−0.30	0.44	RA	0
Belted Kingfisher	4/20	835	−0.01	−2.50 *	−1.25	−0.14	−1.84	−1.34 *	0.09	−1.10	−0.68	WB	0
Yellow-bellied Sapsucker	4/20	596	−0.28	−1.39	−0.33	−0.3 *	−0.92	0.20	−0.23	−1.19	0.17	NE	1.89
Northern Flicker	4/14	1088	−0.29	−0.95	−1.14	−0.05	−1.82	−0.61	−0.11	−1.68	−0.62	BG	0
American Kestrel	4/7	889	0.53 *	−0.87 *	−0.90	0.35 *	−0.61 *	−0.73	0.31	−0.60	0.83	RA	−3.41
Olive-sided Flycatcher	5/24	207	0.16	−0.82	−1.74	0.01	−0.14	−0.12	0.03	0.22	−0.05	AI	0
Eastern Wood-Pewee	5/22	535	0	−1.39	0.24	−0.01	−0.14	−0.15	0.01	0.03	0.30	AI	−3.83
Yellow-bellied Flycatcher	5/23	171	0.31	2.29	1.26	0.29	0.12	0.37	0.31	−1.13	0.03	AI	0
Alder Flycatcher	5/24	376	−0.09	0.08	−0.75	−0.03	−0.14	−0.90	0.13	−0.60	0.14	AI	0
Willow Flycatcher	5/25	210	0	−0.45	0.84	−0.02	−0.32	0.34	0.13	−0.92	0.49	AI	0
Least Flycatcher	5/14	552	−0.13	0.28	0.60	−0.13	−0.50	−0.23	−0.01	−0.86	−0.20	AI	0
Eastern Phoebe	4/10	1139	0.02	−1.29 *	−0.99	0.12	−1.06 *	−1.33 *	0.04	−0.85 *	−1.14 *	AI	0
Great Crested Flycatcher	5/14	810	0.11	−0.25	1.62	0.00	−0.79	0.41	−0.01	−0.93	0.19	AI	0
Eastern Kingbird	5/14	780	−0.07	−0.36	−0.15	−0.10	−0.29	−0.31	0.04	−0.17	−0.32	AI	0
Yellow-throated Vireo	5/19	99	−0.43	−0.46	−0.40	−0.37	−0.32	−0.25	−0.01	−0.28	0.66	LG	0
Blue-headed Vireo	5/3	751	−0.12	−1.00 *	−0.30	−0.20	−0.62	−0.19	−0.11	0.34	−0.02	LG	0
Philadelphia Vireo	5/21	109	0.23	−1.87	−0.19	−0.07	−0.46	0.88	0.06	0.03	0.16	LG	0
Warbling Vireo	5/14	402	−0.21	0.12	0.46	−0.34 *	−0.30	0.27	−0.38 *	−0.71	0.22	LG	0
Red-eyed Vireo	5/18	734	−0.01	−0.79	−0.26	−0.09	−0.51	0.02	−0.13	−0.17	0.17	LG	0
Purple Martin	5/10	143	−0.5	−0.99	0.75	−0.32	0.12	1.45	−0.36	−0.72	0.58	AI	0
Tree Swallow	4/16	1125	0.02	−0.89	−0.70	−0.06	−0.97	−0.42	−0.02	−0.85	−0.64	AI	−4.00
N. Rough-winged Swallow	5/5	316	−0.46	−0.74	−0.25	−0.05	0.29	0.37	0.14	−0.51	0.64	AI	0
Bank Swallow	5/15	271	0.18	−0.01	−0.15	−0.05	−0.19	−0.36	0.15	−0.05	−0.09	AI	−10.83
Cliff Swallow	5/13	318	0.32	0.15	0.09	0.25	−0.95	−0.51	0.41	−0.25	0.04	AI	−2.63
Barn Swallow	5/4	793	−0.15	−0.98	−0.41	−0.13	−0.21	0.11	−0.07	0.01	0.45	AI	−7.90
House Wren	5/8	475	−0.15	−0.67	0.86	−0.14	−0.34	0.27	−0.14	−0.66	0.11	GF	0
Winter Wren	4/24	435	−0.04	−1.49	−0.58	−0.20	−1.3 *	−0.46	−0.05 *	−1.09	−0.41	GF	−6.32
Marsh Wren	5/17	199	−0.12	1.51	0.44	−0.25	0.36	1.05	0.04	−0.01	0.50	GF	0
Blue-gray Gnatcatcher	5/5	198	−0.11	−1.41	−0.62	−0.22	−0.28	−0.54	−0.26	1.06	0.04	LG	0
Ruby-crowned Kinglet	4/23	692	−0.08	−1.02	−0.23	−0.09	−0.76	−0.46	0.07	−0.79	−0.17	LG	0
Eastern Bluebird	4/12	703	−0.86 *	−2.15	−1.07	−0.18	−1.55	−0.99	0.12	−1.31	−0.95	GF	0
Veery	5/15	695	0.21	0.26	0.46	0.03	−1.60	−0.04	0.11	0.01	0.01	GF	−3.83
Swainson’s Thrush	5/19	318	0.28	−0.89	0.41	0.09	−0.57	0.05	−0.03	−0.24	0.03	GF	0
Hermit Thrush	4/24	846	−0.16	−1.41	−0.58	−0.08	−0.71	−0.39	−0.02	−0.52	−0.35	GF	0
Wood Thrush	5/11	667	−0.31	−0.90	15.00	0.00	−0.65	0.42	0.00	−1.07	0.05	GF	−5.62
American Robin	3/21	820	−1.24	−0.13	−1.23	−0.72	−0.86	−0.78	−0.39	−0.78	−0.75	GF	0
Gray Catbird	5/20	1012	−0.19	0.03	0.13	−0.06	−0.25	−0.30	−0.06	−0.28	−0.13	GF	0
Brown Thrasher	5/8	500	−0.21	−1.17	−0.47	−0.16	−0.02	−0.19	−0.12	−0.24	0.12	GF	0
Ovenbird	5/9	978	−0.06	0.15	0.54 *	−0.03	−0.02	0.65 *	−0.08	−23.00	0.21	LG	0
Northern Waterthrush	5/9	451	−0.13	0.16	−0.17	−0.03	−0.07	0.24	−0.03	−0.59	−0.41	LG	−2.15
Black-and-white Warbler	5/6	993	−0.16	−0.11	−0.14	−0.10	0.11	0.25	−0.07	0.26	0.41	LG	0
Tennessee Warbler	5/18	226	0	0.34 *	0.77	0.06	−0.54	0.19	0.11	−0.46	−0.07	LG	0
Nashville Warbler	5/9	662	−0.09	0.24	0.18	−0.11 *	0.36	0.37	−0.13	−0.04	0.15	LG	0
Mourning Warbler	5/25	139	−0.58	−0.22	0.71	−0.10	0.26	0.11	−0.06	0.63	0.39	LG	0
Common Yellowthroat	5/12	972	−0.03	−0.06	0.29	−0.07	−0.12	0.06	−0.08	−0.18	−0.01	LG	−1.77
American Redstart	5/15	823	0.05 *	−0.62 *	0.14	−0.06 *	−0.49 *	−0.06	−0.01 *	−0.3 *	−0.01	LG	0
Cape May Warbler	5/16	175	−0.06	−1.29	−0.55	−0.02	−0.33	−29.00	−0.01	−0.31	−0.13	LG	0
Northern Parula	5/9	896	−0.13	0.45	0.53	−0.12	−0.11	0.54 *	−0.09	−0.44	0.36	LG	0
Magnolia Warbler	5/15	709	0.11	−0.29	0.34	0.00	−0.26	−0.24	0.02	−0.30	−0.22	LG	0
Bay-breasted Warbler	5/18	242	−0.03	−0.09	0.01	0.09	−0.10	0.16	0.06	−0.42	−0.02	LG	0
Blackburnian Warbler	5/15	542	−0.31 *	−0.08	0.29	−0.17 *	0.06	0.10	−0.13	−0.21	−0.05	LG	0
Yellow Warbler	5/11	923	−0.15	0.12	0.35	−0.03	−0.04	0.29	−0.10	−0.02	−0.02	LG	0
Chestnut-sided Warbler	5/13	836	−0.15	−0.46	−0.21	−0.07	−0.09	−0.28	−0.11	−0.09	−0.07	LG	0
Blackpoll Warbler	5/20	391	0.07	−0.45	0.16	0.07	−0.15	−0.01	0.04	−0.36	−0.20	LG	0
Black-throated Blue Warbler	5/12	646	−0.12	0.18	−1.00	−0.11	−0.21	0.07	−0.10	−0.33	−0.01	LG	0
Palm Warbler	4/22	651	−0.14	−0.50	0.34	−0.11	−0.71	−0.08	−0.03	−0.33	0.03	LG	0
Pine Warbler	4/25	675	−0.11	−2.11 *	−0.85	0.00	−1.32 *	−0.40	0.17	−1.13 *	−0.18	LG	4.68
Yellow-rumped Warbler	4/28	1004	0.07	−0.81	−0.09	0.07	−0.53	−0.06	0.06	0.01	0.31	LG	−7.44
Prairie Warbler	5/14	259	0.06	−0.83	0.03	0.09	−0.5 *	−0.26	−0.22 *	−0.36 *	−0.15	LG	0
Black-throated Green Warbler	5/7	988	−0.2 *	0.04	−0.01	−0.13 *	0.02	0.25	−0.13	0.05	0.42	LG	0
Canada Warbler	5/19	464	−0.07	−0.17	−0.02	−0.07	−0.32	−0.05	−0.12	−0.05	0.09	LG	0
Wilson’s Warbler	5/16	409	−0.05	−0.11	−0.20	−0.05	−0.49	0.29	−0.05	−0.42	−0.25	LG	0
Eastern Towhee	5/4	445	−0.03	−0.39	−0.27	−0.17	−0.29	−22.00	−0.15	0.18	0.54	SE	0
Chipping Sparrow	4/21	1097	−0.01	−0.42	0.63	−0.31 *	−1.25 *	0.01	−0.36	−0.85	0.20	SE	0
Field Sparrow	5/4	277	−0.06	−0.84	0.26	0.01	−0.72	0.17	0.11	−0.24	0.05	SE	−6.04
Vesper Sparrow	5/3	180	−0.05	−0.44	0.00	−0.14	−0.10	−0.83	0.32	1.48	−0.19	SE	−3.63
Savannah Sparrow	4/25	659	−0.15	−0.21	−0.80	−0.12	−0.14	−0.33	−0.10	0.30	−0.22	SE	0
Fox Sparrow	3/30	563	−0.17	−1.6 *	−1.2 *	−0.20	−1.32 *	−0.26 *	0.00	−0.83 *	0.15	SE	0
Song Sparrow	3/29	948	−0.28	−1.44	−0.58	−0.23	−1.15	−0.75	−0.08	−1.38 *	−0.72	SE	−2.93
Lincoln’s Sparrow	5/12	253	1.61	−1.60	0.57	0.17	−1.17	−0.19	0.28 *	−1.22	0.14	SE	0
Swamp Sparrow	4/29	500	0.04	−1.61	−0.31	0.05	−0.66	−0.16	0.05	−0.77	−0.22	SE	0
White-throated Sparrow	4/17	979	−1.05	−1.33	−1.81	−0.20	−0.55	−0.15	−0.15 *	0.00	−0.04	SE	−3.89
Scarlet Tanager	5/17	609	−0.07	−0.21	0.22	−0.11	−0.20	0.26	−0.09	−0.30	0.29	LG	0
Rose-breasted Grosbeak	5/10	999	−0.28 *	0.72	0.47	−0.2 *	0.02	0.22	−0.2 *	−0.09	−0.09	SE	0
Indigo Bunting	5/18	528	−0.17	0.69	0.51	−0.24 *	0.44	0.46	−0.27 *	0.50	0.34	SE	0
Bobolink	5/15	766	0.09	−0.29	0.38	−0.01	−0.27	−0.16	−0.01	−0.40	−0.17	SE	−3.28
Red-winged Blackbird	3/22	1251	−0.15	−0.98 *	−0.39	−0.2 *	−0.9 *	−0.48	−0.22 *	−0.84 *	−0.32	GF	0
Eastern Meadowlark	4/24	446	0.07	−0.62	−1.17	0.23	−1.77	−0.82	0.48	−0.22	−0.73	GF	0
Rusty Blackbird	4/11	206	0.23	−2.76	0.61	0.62	−3.19	1.12	0.60	−1.96	1.16	GF	0
Common Grackle	3/24	1207	0.68	−0.75	−0.66	−0.11	0.79 *	−0.57 *	−0.12	−0.81 *	−0.46	SE	0
Baltimore Oriole	5/5	1126	0.06	−1.50	5.89 *	0.05	−2.71 *	3.67 *	−0.07	−1.34 *	1.06 *	GF	0
